# Long-term incidence of relapse and post-kala-azar dermal leishmaniasis after three different visceral leishmaniasis treatment regimens in Bihar, India

**DOI:** 10.1371/journal.pntd.0008429

**Published:** 2020-07-20

**Authors:** Vishal Goyal, Vidya Nand Rabi Das, Shambhu Nath Singh, Ravi Shankar Singh, Krishna Pandey, Neena Verma, Allen Hightower, Suman Rijal, Pradeep Das, Jorge Alvar, Caryn Bern, Fabiana Alves

**Affiliations:** 1 Drugs for Neglected Diseases *initiative* (DND*i*), New York, United States of America; 2 Division of Clinical Medicine, Rajendra Memorial Research Institute of Medical Sciences (RMRI), Patna, Bihar, India; 3 Sadar Hospital Chapra, Saran, Bihar, India; 4 Independent consultant, Bangkok, Thailand; 5 Drugs for Neglected Diseases *initiative* (DND*i*), New Delhi, India; 6 Drugs for Neglected Diseases *initiative* (DND*i*), Geneva, Switzerland; 7 School of Medicine, University of California San Francisco, San Francisco, California, United States of America; Universidade do Estado do Rio de Janeiro, BRAZIL

## Abstract

**Background:**

Few prospective data exist on incidence of post kala-azar dermal leishmaniasis (PKDL) and visceral leishmaniasis (VL) relapse after different treatment regimens.

**Methodology/Principal findings:**

A Phase IV trial included 1761 VL patients treated between 2012–2014 with single dose AmBisome (SDA; N = 891), miltefosine-paromomycin (Milt-PM; n = 512), or AmBisome-miltefosine (AmB-Milt; n = 358). Follow-up for PKDL and VL relapse was scheduled for 6, 12 and 24 months after treatment, lasting until 2017. Patients with lesions consistent with PKDL were tested by rK39 rapid test, and if positive, underwent skin-snip sampling, smear microscopy and PCR. Probable PKDL was defined by consistent lesions and positive rK39; confirmed PKDL required additional positive microscopy or PCR. PKDL and relapse incidence density were calculated by VL treatment and risk factors evaluated in Cox proportional hazards models.

Among 1,750 patients who completed treatment, 79 had relapse and 104 PKDL. Relapse incidence density was 1.58, 2.08 and 0.40 per 1000 person-months for SDA, AmB-Milt and Milt-PM, respectively. PKDL incidence density was 1.29, 1.45 and 2.65 per 1000 person-months for SDA, AmB-Milt and Milt-PM. In multivariable models, patients treated with Milt-PM had lower relapse but higher PKDL incidence than those treated with SDA; AmB-Milt rates were not significantly different from those for SDA. Children <12 years were at higher risk for both outcomes; females had a higher risk of PKDL but not relapse.

**Conclusions/Significance:**

Active surveillance for PKDL and relapse, followed by timely treatment, is essential to sustain the achievements of VL elimination programs in the Indian sub-continent.

## Author summary

Efforts to eliminate visceral leishmaniasis (VL) in the Indian subcontinent have had an unprecedented impact on the number of cases over the past decade, however, the disease is known to be cyclical and previous periods of low incidence have been followed by a major resurgence. The disease is thought to be maintained between epidemics as post-kala-azar dermal leishmaniasis (PKDL), a skin disease affecting 5 to 15% of apparently cured VL patients in the Indian subcontinent. PKDL is difficult to cure and since PKDL patients are not systemically ill, they seldom seek treatment, meaning that PKDL is likely to be a major obstacle to elimination. A key question is how different VL treatment regimens affect the risk of subsequent PKDL. We followed-up a cohort of more than 1700 patients treated with three different drug regimens in an effectiveness trial in Bihar and found that patients treated with miltefosine-paromomycin had a lower risk of relapse but higher PKDL incidence, while AmBisome treated patients had the reverse pattern. In order to sustain the gains already made, active surveillance for PKDL and relapse is essential, along with timely treatment, and new VL treatment regimens that avoid future PKDL are needed.

## Introduction

India has seen an unprecedented decrease in visceral leishmaniasis (VL) incidence over the past decade, with yearly case numbers lower than at any time since the 1970s [1]. This success resulted, at least in part, from the concerted efforts within the kala-azar elimination programme to decrease diagnostic delays and provide effective treatment for VL patients, and integrated vector control. Mathematical models confirm that shortening time from symptom onset to effective treatment should drive the decline below that resulting from the natural incidence cycles of the disease [2]. Previous epidemic cycles in the 1970s and 1990s were followed by intervals of low incidence, but in each instance were followed by a major resurgence [1]. During high incidence periods, kala-azar appears to act as the major infection reservoir, proven to be infectious to sand flies and leading to geographically clustered secondary cases, both symptomatic and asymptomatic [3, 4].

Post-kala-azar dermal leishmaniasis (PKDL) has long been postulated to act as the major inter-epidemic reservoir [5]. PKDL is a pleiomorphic dermatosis that occurs in an estimated 5 to 15% of apparently cured VL patients in the Indian subcontinent [6–8]. Younger age and incomplete treatment with antimonial drugs have been reported to increase risk of PKDL in follow-up data from VL patient cohorts [6, 7]. In longitudinal studies, the median time from VL treatment to PKDL onset has been reported to range from 20 to 36 months, and skin lesions last for years in the absence of treatment [6, 7, 9]. PKDL results in hypopigmented macules, papules, raised indurated nodules or a combination of these. PKDL patients are not systemically ill, and seldom seek treatment. Ascertainment usually requires active case searches, and passive surveillance data greatly underestimate the incidence of disease [6, 9].

Recent xenodiagnosis data confirms that PKDL patients are infectious to sand flies, with higher prevalence of infectiousness among nodular than macular cases [10], supporting the contention that PKDL is likely to prove a major obstacle to sustained VL elimination [8]. Treatment of PKDL is challenging, requiring a prolonged drug course which does not always lead to full resolution of lesions [11, 12]. Avoidance of future PKDL is therefore a high priority in the search for new VL treatment regimens. As the subcontinent transitioned away from antimonial drugs to regimens such as single dose liposomal amphotericin B, researchers expressed optimism that PKDL rates would decrease significantly [13]. However, case reports soon documented PKDL after monotherapy with all of the newer drugs [14, 15].

A key question, therefore, is how different VL treatment regimens affect the risk of subsequent PKDL. Government-mandated first line VL drug regimens have changed over time (first antimonials, then miltefosine, most recently single liposomal amphotericin B), adherence is variable and passive diagnosis of PKDL is highly incomplete. Thus, retrospective analyses may contain inherent biases that impede valid inferences about risk. We undertook prospective follow-up of a cohort of more than 1700 patients treated between August 2012 and October 2014 with three different drug regimens for VL in an effectiveness trial in Bihar [16].

## Methods

### Ethics statement

This protocol was approved by the Institutional Ethics Committee of Rajendra Memorial Research Institute of Medical Sciences (RMRIMS), the Indian Council of Medical Research and National Vector Borne Disease Control Program. All participants provided written informed consent. For children, consent of parents or of a legal representative was obtained.

### Patient population

This analysis presents extended follow-up data from a cohort of participants in a multicenter phase IV clinical trial of three treatment regimens for VL [16]. Patients received treatment for VL in primary health clinics and hospitals in Vaishali and Saran districts, and at RMRIMS between 2012 and 2014. The three VL drug regimens evaluated were (1) single intravenous dose of AmBisome 10 mg/kg (SDA); (2) single intravenous dose of AmBisome 5 mg/kg plus oral miltefosine (AmB-Milt) for 7 days; and (3) intramuscular paromomycin 11 mg/kg plus oral miltefosine for 10 days (Milt-PM). For patients 12 years or older, miltefosine dosing consisted of 100 mg (50 mg twice a day) for those who weighed more than 25 kg, or a single morning dose of 50 mg for those who weighed less than 25 kg. For children 2–11 years, the dose was 2.5 mg/kg/day in two divided doses. Follow up of the patients was done between 2012 to 2017. Patients who had VL relapse or PKDL received rescue therapy following national guidelines.

### Follow-up schedule

The original trial included follow-up visits at 7–20 days and 6 months after treatment [16]. The National Control Program subsequently requested extension to 12 and 24 months of follow-up to assess long-term incidence of VL relapses and occurrence of PKDL. We designated the follow-up visits scheduled at 6, 12 and 24 months as visits 1, 2 and 3. Visits 1 and 2 occurred at the original treatment site for each patient. Visit 3 was conducted at the district hospitals in Saran and Vaishali, and RMRIMS. Study personnel made active attempts to bring back patients who failed to attend scheduled visits.

### Outcomes

Final cure was defined as complete resolution of clinical signs and symptoms of VL. If VL treatment was stopped by the attending clinician for any reason, or the patient failed to finish treatment against medical advice, the outcome was defined as incomplete treatment. The outcome was classified as relapse if the patient had recurrence of VL clinical symptoms after initial clinical recovery and parasites were demonstrated in spleen or bone marrow aspirate. A patient with new onset skin lesions (hypopigmented macules, papules, nodules or a combination) suggestive of PKDL and positive rK39 rapid test was considered to have probable PKDL. Probable PKDL patients were referred to RMRIMS for skin snip sampling, followed by microscopy of the stained skin smear and polymerase chain reaction (PCR) to detect leishmanial DNA. The outcome was defined as confirmed PKDL if the patient had positive results by smear, PCR or both. A patient was considered lost to follow-up if study personnel were unable to trace the patient.

### Data analysis

The sample size was determined by the original trial design, in which 300 patients per arm was calculated to be sufficient for 5% precision around a failure rate of 5% for each arm. Differences between groups were tested using Chi Square test, Fisher Exact test, Wilcoxon Rank Sum, or Kruskal-Wallis tests, as appropriate. We considered the last visit attended by the patient to define total follow-up time, since both study outcomes (relapse and PKDL) were determined at each visit. For incidence density and Kaplan-Meier survival analyses, the censoring date was the last date on which the patient had follow-up, or the date of onset of relapse or of PKDL lesions. Confidence intervals around incidence density were calculated using Poisson regression. Risk factors for relapse and PKDL were assessed in Cox proportional hazard models. Analyses were conducted in SAS 9.4 (SAS Institute, Cary, NC, USA).

## Results

The original effectiveness trial enrolled 1761 VL patients (Table 1). Patients in the AmB-Milt group were significantly older and more likely to be male than those in the other two treatment groups. Eleven patients failed to complete VL treatment, and were excluded from subsequent analyses; all others completed treatment. The median times from treatment to follow-up visits 2 and 3 were 14.1 (interquartile range 12.4–21.6) and 39.4 months (IQR 32.2–44.9) (S1 Table). Of the 1750 patients that completed treatment, 1612 attended follow-up visit 3; for 54 and 66 patients, their last visit was at 6 or 12 months, respectively (S2 Table). Eighteen patients completed treatment and the 7–20 day visit, but failed to attend any subsequent visit; they are included in these analyses, but their contribution in person-time was negligible. Total follow-up time was significantly longer for the group treated with AmB-Milt than the other two treatment groups (median 41.4 months for AmB-Milt versus 36.8 for SDA and 37.3 for Milt-PM; p<0.0001 for both comparisons; NS for SDA vs Milt-PM).

**Table 1:** Demographic characteristics at the time of treatment and completeness of post-treatment follow-up by treatment arm for 1761 visceral leishmaniasis patients, Bihar, India^**1**^.

	VL treatment regimen				
	SDA^2^ (N = 891)	AmB-Milt^3^ (N = 358)	Milt-PM^4^ (N = 512)	p-value	Total (N = 1761)
**Demographics**					
Mean age (years [SD])	24.8 (16.9)	30.4 (17.6)	23.1 (17.8)	< .0001	25.5 (17.5)
Age range (years)	2–80	3–75	2–70		
Age ≤ 12 years N (%)	271 (30.4)	74 (20.7)	189 (36.9)	< .0001	534 (30.3)
Age > 12 years N (%)	620 (69.6)	284 (79.3)	323 (63.1)		1227 (69.7)
Male N (%)	510 (57.2)	247 (69.0)	311 (60.7)	0.0006	1067 (60.6)
					
**Post-treatment follow-up**					
Did not complete VL treatment	4 (0.5)	3 (0.8)	4 (0.8)		11 (0.6)
Lost to follow-up before 6 months	6 (0.7)	9 (2.5)	3 (0.6)		18 (1.0)
Intermediate follow-up^5^	67 (7.5)	32 (8.9)	21 (4.1)		120 (6.8)
Completed last follow-up	814 (91.4)	314 (87.7)	484 (94.5)	0.002^6^	1612 (91.5)
Follow-up time in months					
Median [interquartile range]	36.8 (29.8, 44.4)	41.4 (34.6, 45.1)	37.3 (30.2, 44.0)	0.0001	38.0 (30.4, 44.4)

^1^Treatment occurred between 17 August 2012 and 29 Oct 2014; the last follow-up date was 23 August 2017.

^2^Single dose AmBisome

^3^AmBisome + miltefosine

^4^Miltefosine + paromomycin

^5^Completed 6 and/or 12 month follow-up, but not last follow-up.

^6^P value for comparison of complete vs incomplete follow-up by drug regimen.

A total of 79 relapses and 104 PKDL cases were diagnosed over the course of follow-up. Relapse cases were observed in 47 patients treated with SDA (5.3%), 25 patients treated with AmB-Milt (7%) and in 7 patients treated with Milt-PM (1.4%). PKDL cases were observed in 39 patients treated with SDA (4.8%), 18 patients treated with AmB-Milt (5.7%) and 47 patients treated with Milt-PM (9.7%) (Table 2). Since follow-up times varied significantly by drug regimen, incidence density analysis is a more accurate measure of relapse and PKDL rates in this population. One patient with VL relapse 4.5 months after treatment subsequently presented with PKDL 25.6 months post-treatment; no other patient had both outcomes. PKDL lesions were described as macular (92), maculopapular (10) or papular (2). Seventy patients (67.3%) had confirmed PKDL (36 by microscopy and PCR, 19 by PCR only and 15 by microscopy only) (S3 Table). Thirty-four patients (32.7%) had probable PKDL based on skin lesions consistent with the diagnosis and positive results by rK39 rapid test.

**Table 2:** Incidence of visceral leishmaniasis relapse and post-kala-azar dermal leishmaniasis by treatment regimen among 1750 visceral leishmaniasis patients, Bihar, India.

	VL treatment regimen			
	SDA^1^	AmB-Milt^2^	Milt-PM^3^	Total
Adverse outcomes	N = 887	N = 355	N = 509	N = 1750^4^
**Relapse**				
Person-months of follow-up	29,825.7	12,019.1	17,497.1	59,341.9
Number of patients	47	25	7	79
Incidence density (95% CI)^5^	1.58 (1.18, 2.10)	2.08 (1.41, 3.08)	0.40 (0.19, 0.84)	1.33 (1.07, 1.66)
Time to relapse (months)				
Median [interquartile range]	5.9 [4.1,7.4]	6.6 [4.7,8.1]	11.1 [7.1,13.9]	6.2 [4.5,8.1]
Range	2.0–18.4	3.2–19.8	6.4–20.9	2.0–20.9
**PKDL**				
Person-months of follow-up	30,343.3	12,449.4	17,730.0	60,522.7
Number of patients	39	18	47	104
Incidence density (95% CI)^5^	1.29 (0.94, 1.61)	1.45 (0.91, 2.29)	2.65 (1.99, 3.53)	1.72 (1.42, 2.08)
Time to PKDL (months)				
Median [interquartile range]	23.9 [18.3,32.3]	37.5 [30.3,39.8]	21.4 [12.5,28.3]	25.4 [17.9,33.3]
Range	2.4–48.5	10.8–45.3	1.3–42.8	1.3–48.5

^1^Single dose AmBisome

^2^AmBisome + miltefosine

^3^Miltefosine + paromomycin

^4^Eleven patients who failed to complete VL treatment excluded from analyses

^5^Cases per 1000 person-months of follow-up; CI, confidence interval

**Fig 1:**
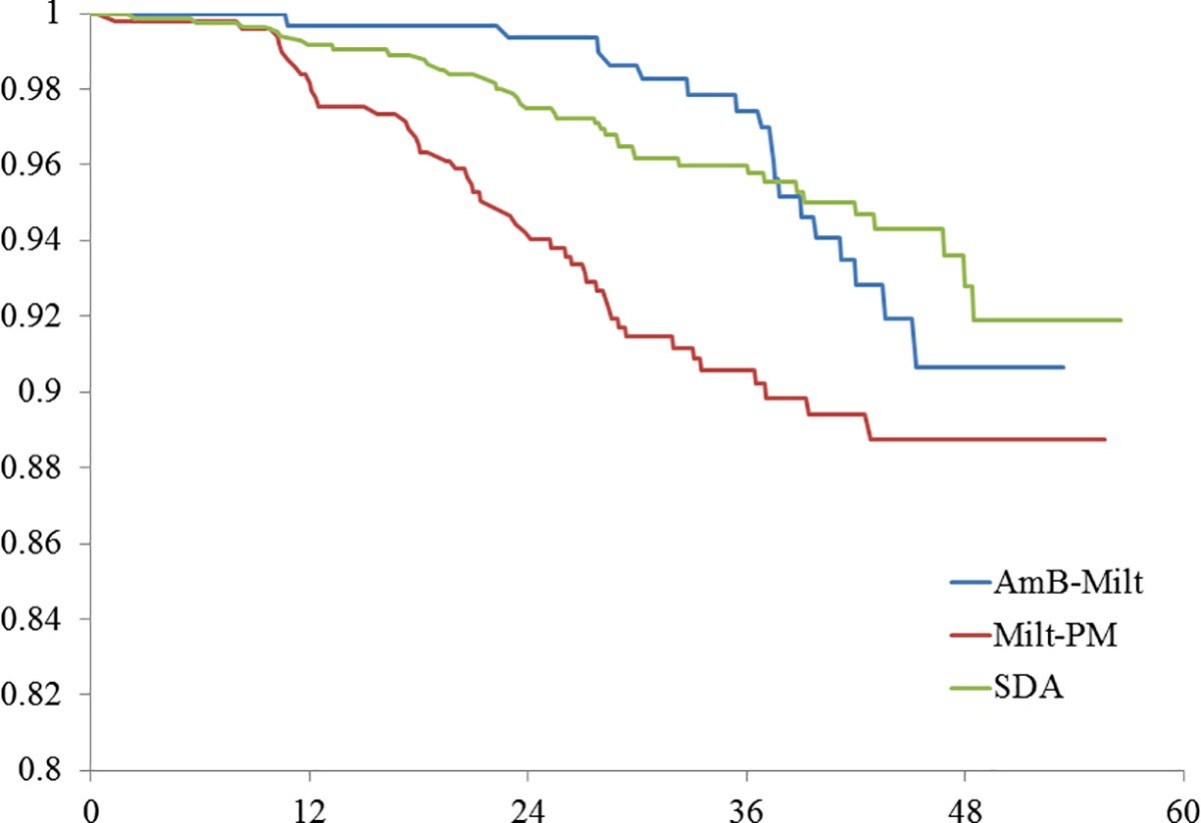
Proportion of study group without PKDL by kala-azar drug regimen by months after treatment. N = 1750. 2 Tail P-value = 0. 0013, Wilcoxon Gehan Test (for early differences); 2 Tail P-value < 0. 0001, Log Rank Test (for later differences).

**Fig 2:**
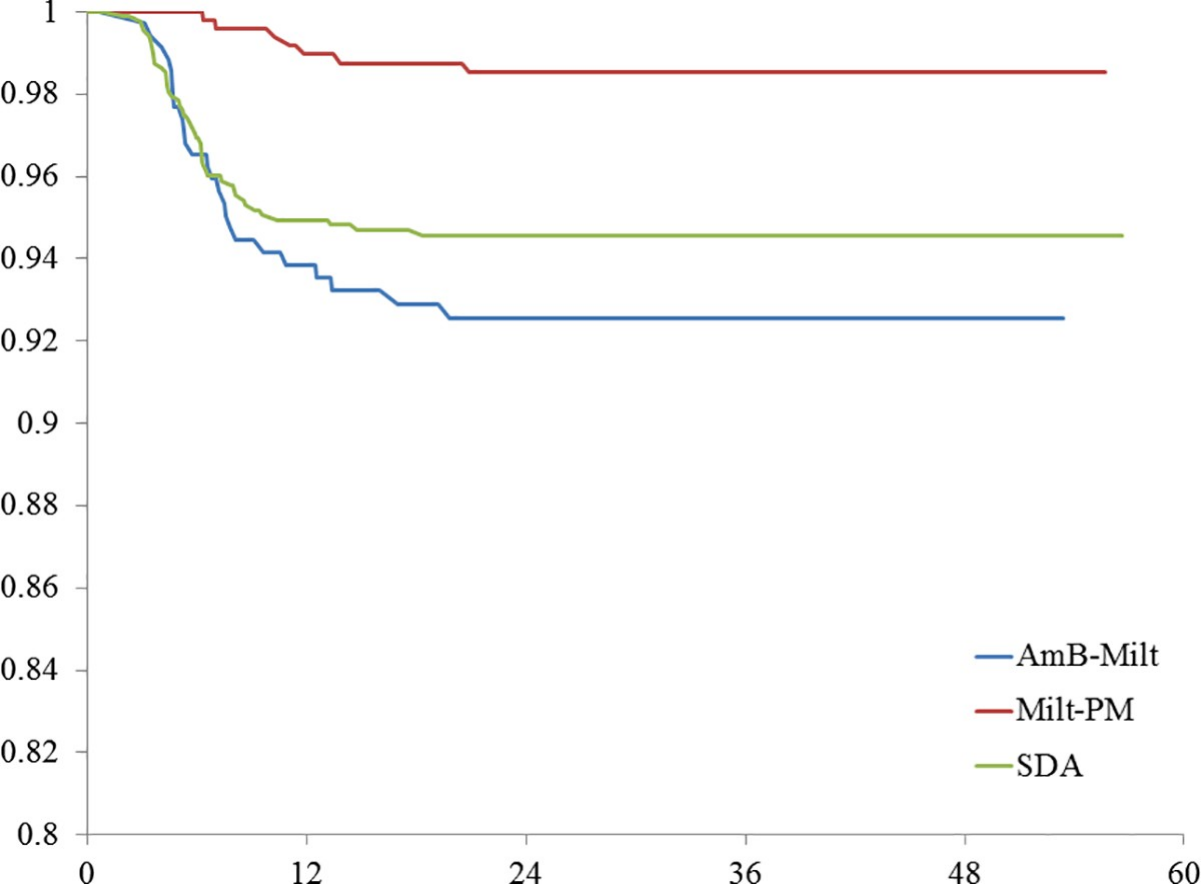
Proportion of study group without relapse by kala-azar drug regimen by months after treatment. N = 1750. 2 Tail P-value < 0. 0001, Wilcoxon Gehan Test (for early differences); 2 Tail P-value < 0. 0001, Log Rank Test (for later differences).

**Fig 3:**
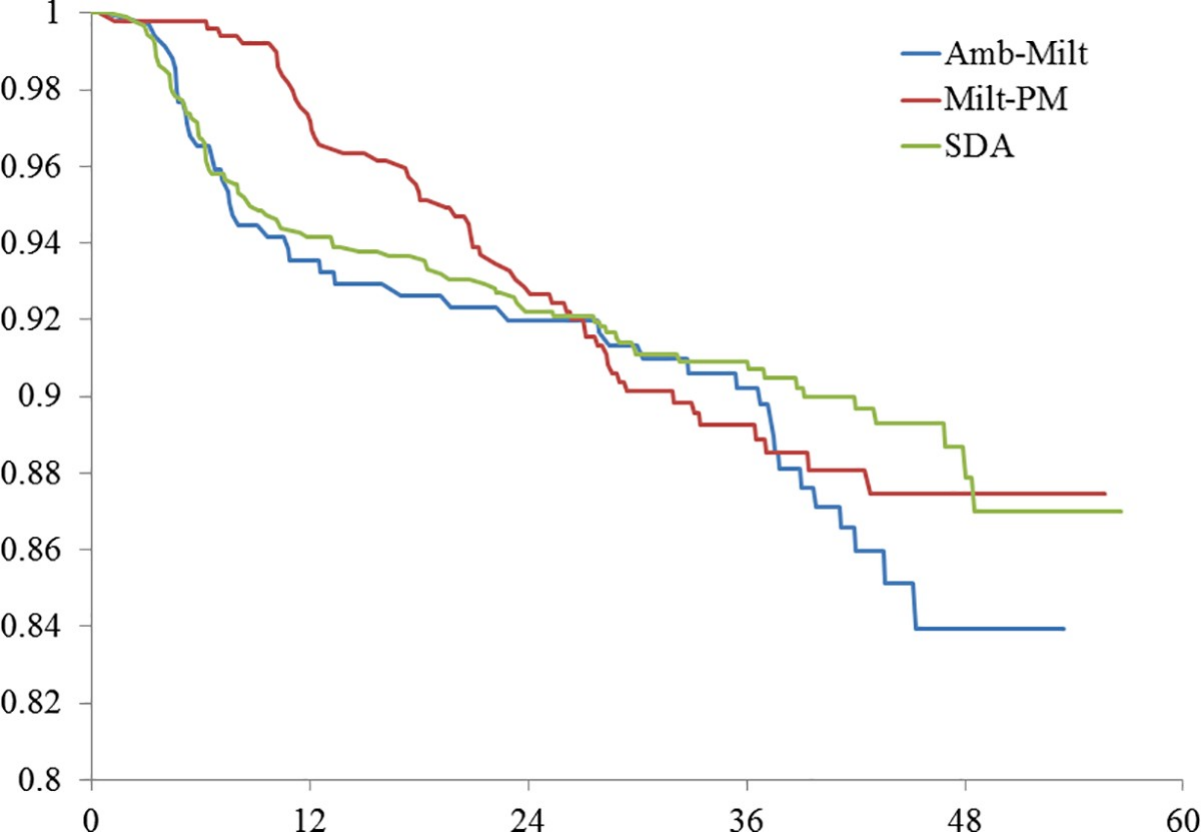
Proportion of study group without either adverse outcome by kala-azar drug regimen by months after treatment. N = 1750. 2 Tail P-value = 0. 7052, Wilcoxon Gehan Test (for early differences.); 2 Tail P-value = 0.5033, Log Rank Test (for later differences).

Relapse incidence density was 1.58, 2.08 and 0.40 per 1000 person-months for SDA, AmB-Milt and Milt-PM, respectively. PKDL incidence density was 1.29, 1.45 and 2.65 per 1000 person-months for SDA, AmB-Milt and Milt-PM. The group treated with Milt-PM had significantly lower relapse but higher PKDL incidence density than the other two groups (Table 2, Figs 1 and 2). The median time to relapse was 6.2 months; 95% of relapses occurred by 17 months of post-treatment follow-up. In contrast, the median time to PKDL lesion onset was 25.4 months and 95% of PKDL cases occurred by 43.1 months post-treatment. When the two adverse outcomes were combined into a single variable (‘treatment failure’), the survival analysis showed no significant difference by regimen (Fig 3).

In univariable analyses, both relapse and PKDL were significantly more frequent among children 12 years or younger, compared to those older than 12 years at the time of VL treatment (Table 3). Relapse was particularly low in VL patients ≥12y treated with Milt-PM. In multivariable Cox proportional hazards models, Milt-PM was associated with significantly lower risk of relapse, but higher risk of PKDL than SDA; rates for Amb-Milt did not differ significantly from those for SDA (Table 4). Age < 12 years was associated with increased risk of both relapse and PKDL, and VL duration longer than 8 weeks prior to treatment was associated with decreased risk of relapse. Females experienced a significantly increased risk of PKDL, but not relapse.

**Table 3:** Incidence of visceral leishmaniasis relapse and post-kala-azar dermal leishmaniasis by age and treatment regimen among 1750 visceral leishmaniasis patients, Bihar, India.

	SDA^1^	AmB-Milt^2^	Milt-PM^3^	Total
**Relapse**				
Age ≤ 12 years (N)	271	73	189	533
Person-months of follow-up	9,076.50	2,485.10	6,255.50	17,817.10
Number of patients	22	8	5	35
Incidence density (95% CI)^4^	2.42 (1.60, 3.68)	3.22 (1.61, 6.44)	0.80 (0.33, 1.92)	1.96 (1.41, 2.74)
Age > 12 years (N)	616	282	319	1217
Person-months of follow-up	20,749.2	9,534	11,241.6	41,524.8
Number of patients	25	17	2	44
Incidence density (95% CI)^4^	1.20 (0.81, 1.78)	1.78 (1.11, 2.87)	0.18 (0.04, 0.71)	1.06 (0.79, 1.42)
**PKDL**				
Age ≤ 12 years (N)	271	73	189	533
Person-months of follow-up	9,328.30	2,583.60	6,374.70	18,286.60
Number of patients	18	6	24	48
Incidence density (95% CI)^4^	1.93 (1.22, 3.06)	2.32 (1.04, 5.17)	3.76 (2.52, 5.62)	2.62 (1.98, 3.48)
Age > 12 years (N)	616	282	319	1217
Person-months of follow-up	21,015	9,865.8	11,355.3	42,236.1
Number of patients	21	12	23	56
Incidence density (95% CI)^4^	1.00 (0.65, 1.53)	1.22 (0.69, 2.14)	2.03 (1.35, 3.05)	1.33 (1.02, 1.72)

^1^Single dose AmBisome

^2^AmBisome + miltefosine

^3^Miltefosine + paromomycin

^4^Cases per 1000 person-months of follow-up; CI, confidence interval

**Table 4:** Risk factors for relapse and post kala-azar dermal leishmaniasis based on Cox proportional hazard models among 1750 visceral leishmaniasis patients, Bihar, India, 2012–2017.

	VL relapse		PKDL	
Characteristic	Hazard ratio (95% CI)	P value	Hazard ratio (95% CI)	P value
VL treatment				
SDA^2^	Referent		Referent	
AmB-Milt^3^	1.39 (0.85, 2.27)	0.1914	1.27 (0.72, 2.22)	0.4115
Milt-PM^4^	0.24 (0.11, 0.54)	0.0005	2.10 (1.37, 3.22)	0.0006
Age				
≤12 years	1.98 (1.26, 3.11)	0.0029	1.85 (1.25, 2.72)	0.002
>12 years	Referent		Referent	
Female	0.77 (0.48, 1.24)	0.2828	1.91 (1.29, 2.81)	0.0011
Male	Referent		Referent	
Illness duration				
<8 weeks	0.28 (0.11, 0.70)	0.0065		
≥8 weeks	Referent			

^1^CI, confidence interval based on multivariable model

^2^Single dose AmBisome

^3^AmBisome + miltefosine

^4^Miltefosine + paromomycin

## Discussion

Twenty years ago, VL patients were almost universally treated with pentavalent antimonial drugs and conventional amphotericin B was largely used as rescue therapy for those failing a prolonged course of pentavalent antimony [17]. Since then, three new drugs, liposomal amphotericin B, miltefosine and paromomycin have been added to the armamentarium and several combination regimens have shown high efficacy for kala-azar treatment [18, 19]. However, there are few valid estimates of PKDL incidence after these newer therapeutic modalities were introduced, and none that include a proper prospective head-to-head comparison among regimens. We extended follow-up after a large effectiveness trial to provide systematic, prospective ascertainment of PKDL after three VL regimens: single dose AmBisome (SDA), AmBisome-miltefosine (AmB-Milt) or miltefosine-paromomycin (Milt-PM).

Our data demonstrate the divergent timing of VL relapses and PKDL. Whereas the median time to VL relapse was 6 months and 95% occurred by 17 months of follow-up, PKDL cases had an onset median of 25.4 months after kala-azar treatment and new cases were still occurring 4 years after treatment. These findings highlight the importance of prolonged follow-up for PKDL after clinical trials, as well as the risk posed by late-occurring cases of PKDL in communities where VL appears to be eliminated. Indeed, further PKDL cases are likely to occur in this patient cohort. The relative timing of relapse vs PKDL in our cohort data is similar to that reported in a recent analysis reporting retrospective and prospective data from participants in several clinical trials in Bangladesh [20]. The divergent study design (hybrid retrospective-prospective with apparent truncation of follow-up at 4 years) and the terms in which incidence was expressed in the Bangladesh analysis (“incidence rate in 100-person-years for 4 years”) impede a direct comparison with our data. Nevertheless, both studies showed a higher incidence of PKDL in those treated with Milt-PM than SDA or AmB-Milt, and both showed lower incidence of relapse for Milt-PM than for the other two regimens.

In common with previous analyses, younger age significantly increased the risk of both PKDL and relapse [6, 21, 22]. Pharmacokinetic studies published subsequent to our trial confirm that adequate miltefosine exposure in children < 12 years requires allometric rather than linear dosing and may contribute to treatment failure in pediatric age groups [23, 24]. However, a widely used AmBisome regimen also showed higher relapse rates in children than adults [21]. Immaturity of the immune system may increase the risk of poor treatment outcomes, independent of drug regimen or dosing.

VL relapse and PKDL risk varied by drug regimen, but interestingly, Milt-PM, the regimen with the lowest relapse rate, was associated with the highest incidence of PKDL, while the two AmBisome-containing regimens, SDA and AmB-Milt, showed the reverse pattern. Further assessment of immune response during and after treatment with these regimens might shed light on the reasons for this pattern, as well as the mechanisms underlying development of PKDL [13, 25]. Ultimately, however, both outcomes represent failures of treatment and the combined incidence of relapse and PKDL was remarkably similar for all three regimens.

Our study faced several crucial challenges and limitations, the most important of which was the difficulty of achieving confirmed diagnoses of PKDL under field conditions in India. We succeeded in confirming two-thirds of the PKDL cases in this analysis by microscopy and/or PCR, while the remaining third was clinically evaluated by a study physician; thus, we believe our case definitions are quite reliable. However, this kind of effort is impossible outside of a funded research study and PKDL diagnosis remains a major challenge in Bihar [26]. In the absence of adequate clinical evaluation, the ‘probable PKDL’ case definition may lead to overdiagnosis, since the rK39 rapid test remains positive for years in cured kala-azar patients [27, 28]. Currently, skin sampling for microscopy is performed at a few tertiary care hospitals and PCR in even fewer centers in Bihar. Upgrading district hospital infrastructure and staff training for skin sampling would enable diagnosis by microscopy at a more peripheral level, and reduce travel costs and lost wages by patients and their families. If sampling and microscopy are performed well, fewer than 30% of patients (19 of 70, in our data) should require referral to a VL reference center for PCR confirmation. However, a long-term solution would require development of non- or minimally invasive sampling methods and a diagnostic technique adapted to field conditions.

Both VL relapse and PKDL are conditions with major public health implications for the sustained control of VL in the Indian subcontinent. Our data demonstrate that we do not yet have an ideal treatment regimen for kala-azar. Clearly, to achieve and sustain kala-azar elimination in the Indian subcontinent, new, preferably oral treatments that are safe and highly efficacious are needed.

## Supporting information

S1 Checklist STROBE checklist. (DOCX)

S1 Table Scheduled and actual follow-up time in months by treatment drug, among 1750 VL patients, Bihar, India, 2012–2017. (DOCX)

S2 Table Follow-up visit attendance by drug regimen among 1750 treated visceral leishmaniasis patients, Bihar, India, 2012–2017. (DOCX)

S3 Table Diagnostic test results for 70 confirmed and 34 probable post-kala-azar dermal leishmaniasis patients, Bihar, India. (DOCX)
