# Supplementary material for: Long-term incidence of relapse and post-kala-azar dermal leishmaniasis after three different visceral leishmaniasis treatment regimens in Bihar, India
Source: PLoS Negl Trop Dis. 2020 Jul 20;14(7):e0008429. doi: 10.1371/journal.pntd.0008429 (PMC7392342; doi:10.1371/journal.pntd.0008429)
Supplement: S1 Table — (DOCX) [file pntd.0008429.s002.docx]

**S1 Table. Scheduled and actual follow-up time in months by treatment drug, among 1750 VL patients, Bihar, India, 2012-2017.**

| **Follow-up visit number**  **(intended timing)** | **Actual follow-up time in months** | | | |  |
| --- | --- | --- | --- | --- | --- |
|  | **All** | **SDA^1^** | **AmB-Milt^2^** | **Milt-PM^3^** | **P value^4^** |
| Visit 1 (6 months) |  |  |  |  |  |
| Mean (SD) | 6.84 (1.27) | 6.70 (1.18) | 6.80 (1.35) | 7.14 (1.31) |  |
| Median (IQR) | 6.4 (6.3, 7.2) | 6.4 (6.3, 6.9) | 6.4 (6.2, 7.0) | 6.7 (6.2, 7.9) | <0.0001 |
| Visit 2 (12 months) |  |  |  |  |  |
| Mean (SD) | 17.17 (6.01) | 15.8 (5.79) | 16.35 (5.00) | 20.56 (5.84) |  |
| Median (IQR) | 14.1 (12.4, 21.6) | 12.9 (12.3, 16.2) | 14.0 (12.4, 19.5) | 21.4 (14.8, 25.3) | <0.0001 |
| Visit 3 (24 months) |  |  |  |  |  |
| Mean (SD) | 38.80 (7.77) | 38.42 (8.16) | 41.33 (6.41) | 37.80 (7.58) |  |
| Median (IQR) | 39.4 (32.2, 44.9) | 38.1 (31.5, 45.1) | 42.3 (37.7, 46.0) | 38.0 (31.2, 44.4) | <0.0001 |

^1^Single dose AmBisome® ; ^2^AmBisome® + miltefosine; ^3^Miltefosine + paromomycin

^4^By Kruskal-Wallis test for comparison among drug treatment groups
