# Supplementary material for: Long-term incidence of relapse and post-kala-azar dermal leishmaniasis after three different visceral leishmaniasis treatment regimens in Bihar, India
Source: PLoS Negl Trop Dis. 2020 Jul 20;14(7):e0008429. doi: 10.1371/journal.pntd.0008429 (PMC7392342; doi:10.1371/journal.pntd.0008429)
Supplement: S2 Table — (DOCX) [file pntd.0008429.s003.docx]

**S2 Table. Follow-up visit attendance by drug regimen among 1750 treated visceral leishmaniasis patients, Bihar, India, 2012-2017.**

| **Follow-up visit attendance** | | | **Drug regimen** | | |  |  |
| --- | --- | --- | --- | --- | --- | --- | --- |
| **6 month** | **12 month** | **24 month** | **SDA^1^** | **AmB-Milt^2^** | **Milt-PM^3^** | **All** | **Censor date** |
| No | No | No | 6 | 9 | 3 | 18 | End of treatment |
| Yes | No | No | 28 | 13 | 13 | 54 | 6m |
| No | Yes | No | 0 | 1 | 0 | 1 | 12m |
| Yes | Yes | No | 39 | 18 | 8 | 65 | 12m |
| No | No | Yes | 16 | 7 | 3 | 26 | 24m |
| No | Yes | Yes | 10 | 5 | 4 | 19 | 24m |
| Yes | No | Yes | 124 | 31 | 127 | 282 | 24m |
| Yes | Yes | Yes | 664 | 271 | 350 | 1285 | 24m |

^1^Single dose AmBisome®; ^2^AmBisome® + miltefosine; ^3^Miltefosine + paromomycin
