# Supplementary material for: Long-term incidence of relapse and post-kala-azar dermal leishmaniasis after three different visceral leishmaniasis treatment regimens in Bihar, India
Source: PLoS Negl Trop Dis. 2020 Jul 20;14(7):e0008429. doi: 10.1371/journal.pntd.0008429 (PMC7392342; doi:10.1371/journal.pntd.0008429)
Supplement: S3 Table — (DOCX) [file pntd.0008429.s004.docx]

**S3 Table. Diagnostic test results for 70 confirmed and 34 probable post-kala-azar dermal leishmaniasis patients, Bihar, India**

|  | | | **VL treatment regimen** | | |  |
| --- | --- | --- | --- | --- | --- | --- |
| **Diagnostic test results** | | | **SDA^1^** | **AmB-Milt^2^** | **Milt-PM^3^** | **Total** |
|  |  |  | N=39 | N=18 | N=47 | N=104 |
| **Confirmed PKDL** | | |  |  |  |  |
| **rK39 RDT** | **Skin smear** | **PCR** |  |  |  |  |
| Positive | Positive | Positive | 16 | 2 | 17 | 35 |
| Positive | Negative | Positive | 4 | 7 | 7 | 18 |
| Positive | Positive | Not done | 3 | 0 | 9 | 12 |
| Negative | Negative | Positive | 0 | 0 | 1 | 1 |
| Negative | Positive | Not done | 1 | 0 | 2 | 3 |
| Negative | Positive | Positive | 0 | 0 | 1 | 1 |
| **Probable PKDL** | | |  |  |  |  |
| **rK39 RDT** | **Smear** | **PCR** |  |  |  |  |
| Positive | Not done | Not done | 6 | 0 | 4 | 10 |
| Positive | Negative | Not done | 1 | 0 | 1 | 2 |
| Positive | Negative | Negative | 8 | 9 | 5 | 22 |

^1^Single dose AmBisome®

^2^AmBisome® + miltefosine

^3^Miltefosine + paromomycin
